# *Haemophilus influenzae:* imposter, mucosal pathobiont, and model organism

**DOI:** 10.1128/jb.00083-26

**Published:** 2026-04-22

**Authors:** Katelyn Amero, Joseph W. St. Geme

**Affiliations:** 1 Department of Pediatrics, Children’s Hospital of Philadelphia and Perelman School of Medicine at the University of Pennsylvania, Philadelphia, Pennsylvania, USA; Dartmouth College Geisel School of Medicine, Hanover, New Hampshire, USA

**Keywords:** *Haemophilus*, mucosal pathogen, pathobiont, model organism

## Abstract

*Haemophilus influenzae* has served as a model organism in numerous ways over the last century. The relationship between *H. influenzae* and the influenza virus provided early insights into bacterial superinfections as a complication of viral infection. Studies of *H. influenzae* have shaped our understanding of virulence mechanisms used by many bacterial pathogens, such as polysaccharide capsules, natural competence for transformation, phase-variable gene expression, and type V protein secretion systems. *H. influenzae* has also been a trailblazer for the development of polysaccharide-protein conjugate vaccines and whole-genome sequencing technologies. In addition, *H. influenzae* has served as a key model organism for mucosal pathobionts, as it is famously associated with both asymptomatic colonization and a variety of disease states.

## INTRODUCTION

“Society does not like an impostor, and when one is found out he is usually despised and mistrusted ever afterwards, whatever merits he may have.”—Turk and May on the reputation of *Haemophilus influenzae* (1).

*Haemophilus influenzae* had a controversial beginning, as it was first isolated from influenza patients and originally believed to be the cause of influenza infection. Upon the discovery of the influenza virus in the 1930s, *H. influenzae* was brushed aside and thought to be clinically irrelevant. Around this same time, *H. influenzae* was confirmed as an asymptomatic colonizer of the human upper respiratory tract (1). Studies of influenza pathogenesis brought *H. influenzae* back into the spotlight and identified bacterial superinfection as a complication of viral respiratory disease, but *H. influenzae* was still underappreciated as a pathogen independent of the influenza virus (1). By the 1960s, *H. influenzae* serotype b (Hib; expressing the serotype b capsule) was clearly recognized as a major pathogen, standing out as the leading cause of bacterial meningitis in early childhood around the world (1). This recognition eventually led to the development of the first polysaccharide-protein conjugate vaccine, which showed superior immunogenicity in young infants and made Hib disease largely preventable (2). With Hib disease becoming a problem of the past, some feel that *H. influenzae* has fallen out of the spotlight again. However, nontypeable *H. influenzae* (NTHi) has regained attention for its role in acute and chronic respiratory tract diseases, as well as its ability to asymptomatically colonize the nasopharynx and cause Hib-like invasive disease (3–6). These diverse properties of NTHi have made *H. influenzae* a model pathobiont.

In addition to its vast clinical relevance, *H. influenzae* has served as a model organism in the laboratory and for many other bacterial pathogens. Studies of *H. influenzae* helped elucidate the role of polysaccharide capsules in bacterial pathogenesis and host immunity (7, 8). They also shaped our understanding of other virulence mechanisms used by bacteria, such as natural competence for transformation (9–11), phase-variable gene expression (12), and type IV and V protein secretion pathways (13–15). Notably, in 1995, *H. influenzae* was the first free-living organism to have its entire genome sequenced (16), making it a pioneer for whole-genome sequencing technologies and tracing microbial evolution.

## “THE IMPOSTER”: EARLY HISTORY AND PATHOGENESIS

The controversial history of *H. influenzae* began with its discovery by Richard Pfeiffer in the early 1890s. Following the 1889 influenza pandemic in Europe, Pfeiffer sought to identify the causative agent of influenza as Pasteur and Koch had recently done with bacterial diseases like anthrax, cholera, and tuberculosis. Pfeiffer looked at purulent sputum collected from patients with influenza and frequently observed small, gram-negative, and poorly staining coccobacilli. He claimed that this organism was not found in control patients and therefore called it “die influenzabacillen” or “the influenza bacillus.” (1, 17, 18) Many were skeptical of Pfeiffer’s conclusions well before influenza virus was discovered in 1933. Other scientists struggled to isolate the bacillus during the subsequent influenza pandemic in 1918, leading to the theory in 1922 that *H. influenzae* was a secondary invader following viral respiratory infection (1, 19). Early studies of influenza in swine demonstrated that inoculation with cell-free mucus filtrate caused mild disease and that inoculation with a *Haemophilus*-like bacterial culture did not cause any disease. It was only when swine were inoculated with both cell-free filtrate and bacterial culture that full-blown influenza disease was recapitulated (1, 20). These simple observations helped uncover the role of bacterial pneumonia as a complication of viral respiratory infections, providing foundational evidence for interactions between viruses and bacteria in disease.

For years, *H. influenzae* was dismissed as “the bacillus that doesn’t cause influenza,” but it was undeniable that this bacterium had an important and complex range of pathogenicity independent from influenza (1). Central to this complexity is the fact that *H. influenzae* violates not one but two of Koch’s postulates, as it can be isolated from healthy individuals, and inoculation of healthy individuals does not necessarily cause disease. Surveys dating back to the 1930s have shown that carriage rates among healthy individuals are as high as 80%, demonstrating that *H. influenzae* is a member of the normal flora in the human upper respiratory tract (1). This observation raised the enduring question of how nonpathogenic strains may differ from pathogenic strains, not only for *H. influenzae* but for all pathobionts associated with both healthy states and disease.

The first distinction established regarding the pathogenicity of *H. influenzae* was between encapsulated (typable) strains and non-encapsulated (nontypeable) strains. In 1931, Margaret Pittman observed that *H. influenzae* strains involved in acute invasive disease tended to have a capsule and a smooth (S) colony morphology, while strains isolated from the upper respiratory tract of healthy people tended to lack capsule and have a rough (R) morphology. Pittman categorized encapsulated *H. influenzae* into serotypes a–f based on the structure of the capsular polysaccharide using agglutination with specific rabbit antisera (1, 21). This categorization revealed that *H. influenzae* serotype b (Hib) was the primary serotype responsible for invasive *H. influenzae* disease, mainly causing meningitis in children under 5 years of age as well as pneumonia, bacteremia, epiglottitis, septic arthritis, osteomyelitis, pericarditis, and cellulitis (1, 22). The unique polyribosylribitol phosphate (PRP) capsule of Hib was also identified as its key virulence factor, protecting the organism against opsonization and phagocytosis and also protecting against desiccation (7, 8). Hib was among the first encapsulated bacterial pathogens described and later gained notoriety as the prototype for polysaccharide-protein conjugate vaccines.

The pathogenicity of *H. influenzae* cannot be fully attributed to its capsule, as non-encapsulated strains are associated with a spectrum of mucosal and invasive diseases. For decades, NTHi was thought to be nonpathogenic or significantly less pathogenic than encapsulated strains, leaving it in the shadow of Hib. NTHi is indeed a component of the normal flora in the human upper respiratory tract, conjunctivae, and genital tract but is also a notable pathobiont with a major disease burden in people of all ages (3–6, 22). It was not until the 1950s that NTHi was identified as a pathogen, first for its role in chronic respiratory infections and exacerbations of chronic obstructive pulmonary disease and bronchiectasis, two highly prevalent conditions (1, 5, 6, 18). NTHi was later identified as one of the most common causes of acute sinusitis and otitis media in all age groups, as well as the main cause of chronic otitis media in children (5, 22). In more recent decades, following the implementation of Hib vaccines, NTHi has gained attention for its ability to cause invasive disease. In countries with high Hib vaccination coverage, NTHi is the leading cause of invasive *H. influenzae* disease and presents as bacteremia without a focus, pneumonia, or meningitis, including in neonates born to mothers who are colonized vaginally with NTHi (3, 5, 6). Incidence rates for invasive NTHi disease are slowly but steadily increasing in these countries, especially in the last decade (3, 6). Recent evidence indicates that NTHi colonization in early infancy may lead to the development of neutrophilic asthma and allergic respiratory disease as well (23). For these reasons, NTHi has now stolen the spotlight from Hib.

As with many pathobionts, understanding which NTHi determinants or host factors are definitively associated with disease remains elusive. This circumstance is mainly due to the genetic and phenotypic heterogeneity of NTHi compared to encapsulated strains, as there are no consistent characteristics found among disease-associated NTHi strains (4, 24). Nasopharyngeal colonization is the only reliable prerequisite for both mucosal and invasive disease and has therefore been the target for vaccines to prevent NTHi infections. NTHi surface structures, such as lipooligosaccharide (LOS) and adhesive proteins, have been extensively studied for their role in colonization, particularly for how they mediate attachment to and invasion of host respiratory epithelia. Research on how these surface structures are recognized by the immune system and how they can be incorporated into an effective NTHi vaccine is ongoing (25–28). As these details of colonization and immunogenicity are being worked out, NTHi is becoming a key model organism for mucosal pathobionts and preventing opportunistic infections at the colonization stage.

## “THE SCAVENGER”: DNA UPTAKE AND NATURAL COMPETENCE FOR TRANSFORMATION

A defining characteristic of *H. influenzae* is its nutritional requirements, not only for erythrocyte factors X (hemin) and V (nicotinamide adenine dinucleotide) but also for nucleotides in general. Upon initial discovery, Pfeiffer quickly learned that adding blood to his culture medium was required for isolation and growth of *H. influenzae*, influencing the genus name *Haemophilus*, referring to blood-loving (1). In the 1950s, Hattie Alexander and Grace Leidy observed that *H. influenzae* could take up and utilize nucleotides from exogenous DNA. They demonstrated this ability by transforming nontypeable (R) strains into typeable (S) strains using DNA-containing fractions from S strains (29). They also conferred streptomycin resistance to susceptible strains using DNA from streptomycin-resistant Hib (30). Natural competence for transformation had already been observed in *Streptococcus pneumoniae*, but these findings in *H. influenzae* provided additional evidence for DNA being the genetic material and for the role of transformation in the evolution of bacterial pathogens (10, 11). Later studies showed that the primary function of DNA uptake in *H. influenzae* is for the recycling of nucleotides from the environment rather than acquiring genetic changes, as most internalized DNA is degraded into nucleotides and used for DNA synthesis. This property is consistent with other fastidious and mucosal organisms, as the bacterium prefers not to synthesize its own nucleotides *de novo* and instead relies on DNA-rich respiratory mucus (31–33).

While *S. pneumoniae* was the first naturally competent bacterium described, *H. influenzae* was found to have a distinct and more efficient mechanism for DNA uptake that relies on sequence specificity (11, 34). In the 1970s, it was observed that *H. influenzae* could only be transformed by homologous DNA, that is, DNA from the same species (35). Subsequent studies demonstrated that it could also be transformed by DNA from its close relative *H. parainfluenzae*, although with reduced efficiency. Interestingly, the efficiency of chromosomal integration for *H. influenzae* and *H. parainfluenzae* DNA was similar, suggesting that *H. influenzae* recognizes self-DNA at the uptake stage (36). These observations led to the discovery of uptake signal sequences (USSs) in some naturally competent bacteria, particularly in *Pasteurellaceae* and *Neisseriaceae*. USSs are 9–12 bp motifs that are enriched throughout the genome and preferred by DNA uptake machinery, appearing at least 600 times in the *H. influenzae* genome and mediating self-DNA uptake bias (9, 10, 34, 37). It was not until the implementation of whole-genome and deep sequencing techniques that these mechanisms were fully characterized. The *H. influenzae* USS is a 9 bp sequence (AAGTGCGGT), with the core tetranucleotide GCGG being critical for uptake. This sequence is flanked by T-rich tracts that are hypothesized to improve DNA uptake efficiency, as the *H. influenzae* genome has a low GC content that may weaken the function of the tetranucleotide core. The *H. influenzae* USS was the first to be identified and characterized in detail for any bacterium, and such methods can now be used to characterize DNA uptake bias in other gram-negative bacteria (38).

*H. influenzae* is an important model organism for studying the mechanisms of competence regulation and transformation. *Escherichia coli* is commonly transformed for recombinant DNA techniques and used as a model for transformation in gram-negative bacteria, but it is not naturally competent like *H. influenzae*. Competence regulation in *H. influenzae* also differs from what was previously described in the gram-positive *S. pneumoniae*. Competence in *S. pneumoniae* is primarily regulated by quorum sensing, while competence in *H. influenzae* is regulated by nutrient signals (10). Early observations showed that competence is enhanced in *H. influenzae* at the end of log phase and that transferring cells from nutrient-rich to nutrient-poor media has a similar effect (39, 40). It is now understood that competence is upregulated in *H. influenzae* when purine nucleotide and other carbon pools are depleted. This depletion increases levels of cAMP, which binds its cofactor cAMP receptor protein (CRP) to induce expression of *sxy*, a gene encoding a competence activator protein. Together, CRP and Sxy induce expression of a 26-gene competence regulon called the CRP-S regulon, which encodes DNA uptake and type IV pilus-like machinery. Following competence induction, double-stranded DNA is imported through the outer membrane, and one strand is degraded as it is translocated across the inner membrane. Nucleotides from the degraded strand are recycled for DNA synthesis, and the other strand is also degraded if it fails to find homology and undergo Rec-dependent recombination. This mechanism is among the best characterized in naturally competent gram-negative bacteria, and the CRP-S regulon is the first to be fully characterized in any organism (9, 11, 33, 41).

Competence and transformation are medically relevant due to their role in facilitating bacterial evolution, and *H. influenzae* has starred in pilot experiments for tracing these phenomena using population genomics. Unlike experimental transformation, genome-wide analyses of *H. influenzae* have allowed investigators to map and quantify genetic variation introduced by transformation. These studies have revealed that transformation is more extensive than originally thought, with some transformant colonies replacing over 3% of their chromosome (42, 43). Recently, a mechanism for the spread of quinolone resistance in *H. influenzae* was defined using genomic techniques (44). Quinolone resistance has been observed in *H. influenzae* worldwide and can now be attributed in part to mutated *gyrA* alleles acquired via horizontal gene transfer. USSs were also identified downstream of *gyrA* and *parC* sequences that confer quinolone resistance, providing additional evidence for antibiotic resistance genes spreading among competent pathogens whose DNA uptake is sequence dependent (44). These approaches may be useful tools for tracing the spread of genetic traits in nature and among clinical isolates and also for understanding the general mechanisms of horizontal gene transfer in bacteria.

## “THE PROTOTYPE” PART I: POLYSACCHARIDE-PROTEIN CONJUGATE VACCINES

Before the development of the first polysaccharide-protein conjugate vaccine, Hib was a leading cause of bacterial meningitis and mortality in children under 5 years of age. It accounted for 95% of invasive *H. influenzae* disease in children and over half of invasive *H. influenzae* disease in adults, causing up to 30,000 cases a year in the US in the 1980s (22, 45). For Hib meningitis, mortality rates are as high as 10%, and permanent neurological sequelae develop in 30% of survivors (46). Development of an effective Hib vaccine became a major priority as other now-routine vaccines were being developed. However, achieving successful immunization in the target population of infants and young children proved to be a unique challenge.

As with other encapsulated bacteria like *S. pneumoniae* and *Neisseria meningitidis*, the capsular polysaccharide of Hib is highly immunogenic and can stimulate protection against infection. It was demonstrated back in 1940 that immunizing animals with Hib elicited anti-PRP antibody responses (47). However, it was not until 1972 that anti-PRP antibodies were characterized in humans and were elicited experimentally by immunization with purified PRP (48, 49). This early immunization study showed that PRP had comparable immunogenicity to the capsular polysaccharides of *S. pneumoniae* and *N. meningitidis*, although a key limitation was that this initial study was only conducted in adult volunteers (49). A larger-scale, double-blinded study was conducted in Finland in the mid-1970s and included approximately 100,000 participants aged 3 months to 5 years. Half of the participants received the purified PRP vaccine, while the other half received a meningococcal vaccine as a control. In the first year of follow-up, there were 11 cases of invasive Hib disease in the control group and zero cases in the PRP group among children aged 18 months or older. In children younger than 18 months, no protection was observed, and anti-PRP antibody responses were poor even after a booster dose was administered (50). These results were confirmed in subsequent analyses of Finnish studies, which calculated a 90% efficacy rate in children older than 18 months. Combined with a vaccination coverage of 90%, the original PRP vaccine could have prevented around 50% of all invasive *H. influenzae* cases (46).

The lack of efficacy in children younger than 18 months was a major limitation of the original PRP vaccine and warranted a novel vaccine design. Hib meningitis was most prevalent during the first 2 years of life, starting at a few months of age as maternally acquired antibodies start to decrease (18). Therefore, it was crucial that the Hib vaccine was modified for efficacy in young infants. Bacterial polysaccharides, in general, are poor immunogens for young infants because they are T-cell independent antigens and rely on antibody responses that are immature in early life (18, 50). To overcome this limitation, researchers at the NIH considered an idea described in 1929, conjugating carbohydrates to protein carriers for boosting immunogenicity (51, 52). Conjugated PRP had already been shown to be immunogenic in infants, to generate high levels of IgG, and to produce booster responses (53–56). The first PRP conjugate vaccine tested for protective efficacy was designed using the diphtheria toxoid as the protein carrier and was called the PRP-D vaccine. In a 1987 Finnish study of approximately 30,000 infants aged 3–14 months, the protective efficacy of the PRP-D vaccine was 83% after 3 doses at 2, 4, and 6 months (57). While this result was a significant improvement from the plain PRP vaccine, it still required optimization to ensure sufficient protection. Other protein carriers that were tested included a genetically engineered diphtheria toxoid (PRP-CRM197), *N. meningitidis* outer membrane proteins (PRP-OMP), and the tetanus toxoid (PRP-T). Results with the PRP-T vaccine were especially striking, showing a staggering 100% efficacy among 100,000 Finnish children who received two or more doses (2). In the early 1990s, the PRP-T vaccine was licensed for infants around the globe, starting at 2 months of age.

Not only were the Hib conjugate vaccines an immense success in reducing Hib disease, but they also served as a model for other encapsulated bacterial pathogens. Before the PRP and PRP-T vaccines, the incidence of Hib meningitis was 20–50 cases per 100,000 children under 5 years old in the United States and Europe (58). As of 2017, the incidence of all invasive Hib disease in the United States was 0.18 per 100,000 children under 5 years of age, a greater than 99% decline (22). The marked efficacy of these Hib conjugate vaccines inspired the development of improved vaccines for *S. pneumoniae* and *N. meningitidis*, both of which were licensed in the United Kingdom in 1999 and in the United States in 2000. More recently, a *Salmonella enterica* serovar Typhi conjugate vaccine was licensed in India in 2017, and conjugate vaccines have also been developed for *Salmonella enterica* serovar Paratyphi*, Shigella*, and group B *Streptococcus* (59).

## “THE OPPORTUNIST”: PHASE VARIATION AND WHOLE-GENOME SEQUENCING

In the 1980s, an interesting observation was made regarding capsule expression by Hib. It was already understood that capsule expression was an unstable feature of *H. influenzae*, but the mechanism and regulation of this phenomenon were unclear. Eventually, Richard Moxon and colleagues noticed that the loss of capsule expression in Hib occurred spontaneously, both *in vitro* and in an infant rat model of invasive disease. These investigators also noticed that capsule-deficient variants lacked an EcoRI restriction fragment when probed with a cloned piece of DNA containing sequences known to be necessary for capsule expression, suggesting that capsule expression can be switched off by a specific recombination event (60). They later established that capsule loss occurs through Rec-dependent recombination at a 17 kb tandem duplication of the *cap* locus (61). This duplication flanks a bridge region that contains a gene required for capsular polysaccharide export called *bexA*. Upon recombination, one copy of the duplication and the only copy of *bexA* are lost, switching off capsule production (62). This observation was the first time that phase variation was described in *H. influenzae*. Subsequent studies demonstrated that the duplicated arrangement of the *cap* locus can also result in amplification of capsule gene sequences, leading to increased PRP production through a gene dosage effect (8, 63).

Phase variation in *H. influenzae* is far from limited to the recombination mechanism in capsular polysaccharide expression. Similar observations were made regarding the expression patterns of LOS and pili, but their molecular mechanisms are quite distinct. LOS appeared to be phase variable when it was observed that anti-LOS monoclonal antibodies frequently gain and lose reactivity (64). This mechanism involves 4 bp (CAAT) tandem repeats in the open reading frames of several LOS biosynthesis genes. The number of repeats varies due to slipped-strand mispairing during DNA replication, causing spontaneous frameshift mutations that turn expression on or off. There are at least seven known phase variable genes involved in LOS post-translational modification, most of which are in the *lic* loci (*lic1*, *lic2*, and *lic3*) (65–68). Similar mechanisms were previously described for *Neisseria* spp., but a completely novel mechanism of phase variation was described for *H. influenzae* pili a short time later. Like capsule expression, pili expression occurs in three levels (−/+/+++) but is instead controlled by two divergently transcribed genes, *hifA* and *hifB*. The *hifA* and *hifB* promoters have significant overlap and contain 2 bp (TA) tandem repeats. Variation in these repeats shifts the spacing of the −35 and −10 promoter elements and alters transcription of *hifA* and *hifB* simultaneously. This finding was both a novel mechanism of gene regulation and the first description of intrastrain pili variation (69). These two discoveries were also among the first descriptions of repeat sequences regulating gene expression in prokaryotes, resembling microsatellite sequences in eukaryotic DNA. *E. coli* has been used to study these repeat sequences, but they are far more abundant and functional in the *H. influenzae* genome (70, 71).

The search for additional phase variable genes was pivotal to the selection of *H. influenzae* strain Rd as the first free-living organism to have its entire genome sequenced and assembled in 1995 (16). This sequencing project was a milestone of its own, but it also revealed the true prevalence and diversity of phase variation mechanisms in the *H. influenzae* genome. Once this sequence was available, it was probed for tandem repeat sequences, and at least 14 phase variable genes were initially identified in strain Rd and other strains (72, 73). Most of these genes have 4 bp tandem repeats, but not all. For example, there are 7 bp (ATCTTTC) repeats in the promoters of *hmw1A* and *hmw2A*, which encode the HMW1 and HMW2 high molecular weight adhesins involved in NTHi colonization. The mechanism of HMW1 and HMW2 phase variation was also novel at the time of discovery. As the number of repeats increases, HMW expression decreases in a stepwise manner rather than switching between on/off states or three expression levels (74). A similar mechanism has been observed more recently for the *hmw1C* and *hmw2C* genes in the *hmw1* and *hmw2* loci, encoding glycosyltransferases that modify the HMW1 and HMW2 adhesins. As the number of 9 bp (CAAACCAAG) repeats increases, glycosyltransferase expression and HMW1 and HMW2 glycosylation experience a stepwise decrease (75). It is still unclear how these repeats alter gene expression at the transcriptional or translational level. Regardless, *H. influenzae* was the first organism identified to have several phase variable genes with entirely distinct mechanisms. It is now an important model for the use of whole-genome sequencing to identify nucleotide repeat sequences and phase variable genes in other organisms, such as *N. meningitidis*, *Campylobacter jejuni*, and *Helicobacter pylori* (12, 72).

Phase variable genes in *H. influenzae* encode primarily cell surface structures, but the sequence of *H. influenzae* Rd revealed a distinct and perplexing gene that is subject to phase variation. Tetranucleotide (AGTC) repeats were identified in the open reading frame of the *modA* gene, which encodes a DNA methyltransferase with homology to those of type III restriction-modification (R-M) systems (70, 72). Phase variation had never been described in R-M systems, and it was initially thought that phase variation functions to modulate phage defense. The type I R-M system of *H. influenzae* is more active in phage defense and was also found to be phase variable via 5 bp repeats. Studies of phase variation in this R-M system revealed that changes in phage susceptibility were more heavily tied to the phase variation of LOS, a common receptor for *H. influenzae* phage (76). The function of the ModA methyltransferase in phase variation proved to be much more interesting and complex than phage defense alone. When expression is on, ModA targets other genes for methylation to regulate their expression, like epigenetic modification in eukaryotes. Following this discovery, regulons of genes subject to phase-variable methylation were named “phasevarions.” The ModA system was one of the first examples of epigenetic gene regulation in prokaryotes, and phasevarions have now been identified in many other bacterial species as an additional mechanism of phase variation (77).

Despite the technical difficulties of studying phase variation, some of its roles in pathogenicity and disease have been characterized in *H. influenzae*. For example, when the gene duplication is intact in the *cap* locus of Hib, PRP expression is doubled and may increase immune evasion and invasiveness. Production of excess PRP may also help saturate host opsonization factors as PRP is released from the bacterial surface (8, 63). Phase variable modification of LOS has been shown to increase virulence *in vivo*, as modified LOS escapes multiple arms of host immunity. Acetylation and various carbohydrate modifications prevent opsonization by both steric hindrance and molecular mimicry of host cell structures (64, 78, 79). Genetic analyses of clinical NTHi isolates suggest that these modifications are off during asymptomatic carriage but on during invasion (68). The opposite pattern has been observed with pili and the HMW1 and HMW2 adhesins. During nasopharyngeal carriage, NTHi expression of pili and the HMW1 and HMW2 adhesins is generally on, consistent with the roles of these adhesins in colonization. During spread from the nasopharynx, pili expression is off, and HMW1 and HMW2 expression is reduced, reflecting selection for variants that are able to escape antibody responses (69, 74, 80). For the HMW adhesins, the role of phase variation may be more complex, as phase variable glycosylation may impact NTHi colonization and immune evasion.

## “THE PROTOTYPE” PART II: TYPE IV AND V SECRETION SYSTEMS

Before the discovery of a novel *H. influenzae* type IV secretion system, *H. influenzae* was already a model bacterium for type V secretion. Type V secretion systems can be divided into subtypes Va through Ve (81), with Va, Vb, and Vc having been identified and well characterized in *H. influenzae*. The type Va and Vc protein secretion systems are also called autotransporters because they do not use an energy source and were initially believed to be fully self-sufficient systems for secretion across the outer membrane (82). It is now recognized that these proteins are secreted by the β-barrel assembly machine complex (83). Autotransporter proteins are exclusively found in the outer membrane of gram-negative bacteria and carry out a variety of effector functions, including adherence, invasion, cytotoxicity, and protease activity (84, 85).

Type Va-secreted proteins are considered the classical autotransporters, and examples in *H. influenzae* include IgA1 protease and Hap. These proteins have a common structure consisting of a signal sequence at the N-terminus for Sec-dependent translocation across the inner membrane, a 12-stranded β-barrel at the C-terminus that forms a translocator domain in the outer membrane, and an internal passenger domain that is presented on the bacterial surface (13, 81). The *Neisseria* spp. IgA1 protease is among the best characterized classical autotransporters and shares considerable homology with the *H. influenzae* IgA1 protease and Hap proteins (13, 86). The *Neisseria* spp. and *H. influenzae* IgA1 proteases function to cleave human IgA1 and presumably promote colonization, as IgA1 agglutinates bacteria at mucosal surfaces like the nasopharynx (13, 87). The Hap autotransporter is a serine protease like the IgA1 proteases and mediates autoproteolytic cleavage, releasing the Hap passenger domain from the bacterial surface (13, 88, 89). In the human upper airway, Hap autoproteolysis is inhibited by secretory leukocyte protease inhibitor, which normally functions to protect the epithelium from inflammatory proteases (90, 91). Inhibition of Hap autoproteolysis results in retention of the Hap passenger domain on the bacterial surface, facilitating adhesive activity, which resides in the passenger domain. When Hap is uncleaved, and the passenger domain remains on the bacterial surface, Hap facilitates colonization by mediating adherence to human epithelial cells and extracellular matrix proteins and by promoting bacterial aggregation and microcolony formation (13, 91, 92). Conversely, release of the Hap passenger domain may allow *H. influenzae* to migrate from the upper airway following colonization.

Type Vc-secreted proteins are similar in structure to the type Va-secreted proteins, except they are trimeric rather than monomeric autotransporters. In the trimeric autotransporters, each monomer contributes 4 β-strands to form a 12-stranded β-barrel translocator domain in the outer membrane, analogous to the 12-stranded β-barrel domain in classical autotransporters. Each monomer also contributes its own passenger domain, together forming an intertwined structure with a coiled coil stalk and an N-terminal globular head (13, 81, 93, 94). The NTHi Hia protein is a prototype trimeric autotransporter that mediates high-affinity adherence to respiratory epithelial cells and contains two binding pockets on each face of the trimer. The *H. influenzae* Hsf trimeric autotransporter is present in all typeable strains and is highly homologous to Hia, with identical binding pockets, but is longer, allowing it to extend beyond the polysaccharide capsule (13, 95, 96).

The *H. influenzae* HMW1 and HMW2 adhesins are prototype type Vb-secreted proteins (14). Type Vb-secreted proteins contain two separate polypeptides encoded by a single operon; hence, they are also called two-partner secretion (TPS) systems. The two proteins include an outer membrane translocator (TpsB) and a secreted protein (TpsA). The N terminus of TpsA proteins contains a signal sequence for Sec-dependent translocation across the inner membrane, as well as a TPS secretion domain that targets it to its cognate TpsB protein. TpsB proteins have a 16-stranded β-barrel structure with two polypeptide transport-associated (POTRA) domains facing the periplasm. These POTRA domains interact with the TPS secretion domains of TpsA proteins and facilitate their export across the outer membrane via the TpsB pore (81, 97, 98). The fate of TpsA proteins is either to be released from the organism or remain non-covalently bound to the bacterial surface, with the latter being true for HMW1 and HMW2. HMW1 and HMW2 are the TpsA proteins encoded by the *hmw1* and *hmw2* loci, respectively, and form disulfide bonds via C-terminal cysteine residues to tether themselves to their TpsB proteins, HMW1B and HMW2B (14, 99). Other TpsA proteins, such as ShlA of *Serratia marcescens*, lack these C-terminal cysteine residues and are therefore released extracellularly (81, 100). Additionally, some TPS systems contain a third accessory gene encoding a glycosyltransferase, with the HMW1C and HMW2C proteins being prime examples. Glycosylation is crucial to the stability, tethering, and cellular adherence properties of HMW1 and HMW2 (101). Similar accessory genes have been identified in TPS systems in *Actinobacillus pleuropneumoniae*, *Yersinia enterocolitica*, and *Yersinia pestis* (14).

In addition to being a model bacterium for type V secretion, *H. influenzae* is a model for an unusual type IV secretion system. Type IV secretion systems conventionally deliver bacterial effector proteins into host cells and facilitate horizontal gene transfer via conjugation in both gram-negative and gram-positive bacteria. Previously described lineages of type IV secretion systems have been categorized into subtypes F, P, and I based on their conjugative plasmids. In 2007, a unique and evolutionarily distant lineage was identified on an *H. influenzae* genomic island called ICE*Hin1056*, which is a known antibiotic resistance vector. Rather than propagate effector proteins or conjugative plasmids, this type IV secretion system helps propagate the genomic island on which it is encoded and is positively regulated by one of its own gene products (15). Similar type IV secretion systems have now been identified in a subset of *Pasteurellaceae* species (102, 103).

## CURRENT QUESTIONS AND FUTURE DIRECTIONS

There are many important questions, both old and new, to address regarding *H. influenzae* biology and disease. Related to competence, a USS-binding protein for mediating sequence-specific DNA binding at the cell surface has yet to be identified in *H. influenzae* (38). Also uncharacterized is the mechanism for the uptake of donor DNA. A “competence pseudopilus” related to type IV pili and type II secretion systems has been implied in *H. influenzae*, other gram-negative bacteria, and some gram-positive bacteria but has not been observed at any bacterial surface (9–11, 41). The membrane-bound endonuclease responsible for degrading one strand of incoming duplex DNA has not been identified either, although it seems to have substrate requirements like those of Hind nucleases (104). Elucidating these structures and mechanisms for DNA uptake and processing would be a major development in understanding competence in both gram-negative and gram-positive bacteria.

Whole-genome sequencing techniques pioneered in *H. influenzae* have opened doors for identifying competent bacteria and tracing bacterial evolution. The distribution of natural competence in bacteria is likely underestimated because the conditions that make them competent are also unknown. Sequencing approaches developed with *H. influenzae* can be applied to other organisms to trace the acquisition of new DNA elements, determine if there is uptake bias for self-DNA, and characterize DNA uptake specificity (38, 42, 43). These approaches have also allowed researchers to trace horizontal gene transfer and the evolution of microbes in nature. This is an incredibly useful tool in surveillance for the spread of antibiotic resistance genes in bacteria and the emergence of novel pathogens, two increasingly frequent phenomena (44, 105).

As focus has shifted away from Hib and toward NTHi, many novel questions regarding immunogenicity and vaccine design have emerged. Since NTHi lacks a capsular polysaccharide, the Hib conjugate vaccine platform cannot be utilized in an NTHi vaccine. Another major barrier for developing an NTHi vaccine is the extensive inter- and intrastrain heterogeneity within singular hosts, especially with respect to their surface structures. It is unclear exactly how these surface structures contribute to disease, but many of them are implicated in nasopharyngeal colonization, a prerequisite for disease. Therefore, the aim of an NTHi vaccine would be to prevent or reduce colonization. Potential vaccine antigens include LOS and the HMW1 and HMW2 adhesins due to their surface localization and immunogenicity, but their incorporation in a vaccine is complicated by their phase variability. Protective immune responses have been observed in several animal models using these antigens, but where protective antibodies bind and how they function in the context of phase variable modifications is still unknown (25–28). Some investigators believe that phase variable proteins should not be incorporated into vaccines, but the Men4B vaccine is a key example of phase variable proteins being used in successful multicomponent vaccines. The “evolutionary trap” idea also makes a good case for these vaccines, stating that escape from vaccination-induced immunity should require the pathogen to lose or compromise that virulence characteristic (12). Additional research is required to understand how phase variation in NTHi surface structures impacts virulence and immunity before they can be incorporated into a vaccine.

Since the early 20th century, the same general questions remain regarding *H. influenzae*: What makes commensal strains different from pathogenic strains? Do commensal strains become pathogenic strains? If so, how? While a few pieces of the puzzle have been put together, the big picture has not been resolved. Hib has been the more notorious pathogen and the focus of most *H. influenzae* research, but NTHi is the true pathobiont that has given rise to these fundamental questions and now demands attention. NTHi is associated with asymptomatic carriage and mucosal disease in people of all ages and also causes Hib-like invasive disease in both the very young and the elderly. Current research efforts should aim to further connect the diverse genetic, structural, and immunogenic characteristics of NTHi with its diseases. This knowledge could help make the vast majority of *H. influenzae* disease preventable and a problem of the past.
